# Individual and School Environment Predictors of Mental Health and Wellbeing Across the Primary-to-Secondary School Transition

**DOI:** 10.1007/s12310-025-09776-9

**Published:** 2025-06-25

**Authors:** Caitlyn Donaldson, Jemma Hawkins, Graham Moore

**Affiliations:** 1 Centre for Development, Evaluation, Complexity and Implementation in Public Health Improvement (DECIPHer), Cardiff University, Cardiff, UK; 2 Wolfson Centre for Young People’s Mental Health, Cardiff University, Cardiff, UK

**Keywords:** Mental health, Mental wellbeing, School transition, Socioeconomic status, Meaningfulness

## Abstract

Transition from primary to secondary school is an important life event for young people that may have impacts on mental health. Objective and subjective measures of the school environment may be associated with mental health outcomes post-transition. Pre-transition (year 6, aged 10–11) survey data from young people in Wales, UK, were linked to post-transition (year 7, aged 11–12) survey data (*n* = 506) and combined with an objective value-added measure of school meaningfulness created from administrative data. Multi-level models were run, adjusting for pre-transition mental health difficulties, to investigate the relationship between individual and school-level variables, including self-reported perceptions of school connectedness and school meaningfulness, and mental health difficulties and mental wellbeing outcomes post-transition. Pre-transition mental health difficulties were consistently significantly associated with post-transition mental health difficulties. Higher family affluence was significantly associated with higher mental wellbeing and lower difficulties. School connectedness items also showed evidence of significant association with mental health difficulties and wellbeing outcomes post-transition. School meaningfulness was only significantly associated with post-transition mental wellbeing. Individual- and school-level factors offer opportunities for targeting interventions to support young people’s mental health and wellbeing across the school transition period.

**Supplementary Information:**

The online version contains supplementary material available at 10.1007/s12310-025-09776-9.

## Introduction

The transition from primary to secondary school is an important life event for young people. While for many it is a positive experience that is perceived as an exciting challenge, for others it is viewed as a threat to their mental health (Sirsch, 2003). Mental health can be considered as including both positive and negative aspects. On the negative side, mental health difficulties may include symptoms of depression, anxiety and aggression, while mental wellbeing focuses on positive assets, such as life satisfaction, purpose in life, positive relationships with others and self-actualisation (Keyes, 2005; Ryff, 2013). This paper explores how factors at the individual and school level might be associated with the positive and negative aspects of young people’s mental health, across the primary-to-secondary school transition.

The impact that transition has on a young person’s mental health is a function of both their own personal resources and the school environment that they are transitioning into (Donaldson et al., 2023a). Young people with higher psychosocial resources (self-esteem, self-efficacy, social support) and greater access to flexible resources (money, power, cultural capital) (Link & Phelan, 2010; Taylor & Broffman, 2011) may be better able to adapt and adjust to the challenge of transition. There is evidence that young people who are worried about transition have higher emotional difficulties and loneliness post-transition (Moore et al., 2021; Rice et al., 2021). Concurrently, the school environment, if it aligns culturally to that of the young person, may support a less stressful transition experience, reducing the personal resources needed. Creating a school environment matched to the needs of the young person is therefore particularly relevant for those who have lower resources, for example, those with low socioeconomic status (SES) (Oakes & Rossi, 2003).

There are objective and subjective measures of school environment. Objective measures are based on school data, such as school size and physical amenities, discipline policies and pupil-to-teacher ratio (Bonell et al., 2013). They also include the percentage of students eligible for free school meals (FSM) as a measure of school affluence, the age-range of students within the school and the policies and processes associated with transition. Subjective measures are those based on the perceptions and beliefs of students, teachers and parents about the school.

### Objective Measures of the School Environment: Value-Added Education

In addition to those highlighted above, measures of value-added education provide an interesting perspective on school environment. They have typically been used as a means of assessing school performance by measuring student academic outcomes adjusted for student demographic background (Leckie & Goldstein, 2019). However, they have also been used within a health context to explore how value-added education relates to health-related behaviours like smoking and substance use (e.g. Aveyard et al., 2004; Bonell et al., 2017).

The value-added measure used within these studies uses two objective outcomes—attendance and attainment—and then constructs a model that seeks to predict these two outcomes based on the demographic characteristics of a school. Where schools are doing better in terms of their attendance and attainment than would be predicted based on their demographics, they have a positive residual value (i.e. their true value is above the line of best fit), where schools are doing worse in terms of their attendance and attainment than would be predicted based on their demographics, they have a negative residual value (i.e. their true value is below the line of best fit). Schools with a positive residual are adding value to their students by producing better outcomes than would be predicted based on the school’s demographics alone.

Markham (2015), however, takes this further, by suggesting that the value-added measure can be used as a proxy for a subjective experience—whether or not schools are perceived to be meaningful by their students. School meaningfulness describes whether a school’s aspirations and aims for its students align with those of the student and the community from which they come, and therefore the extent to which a school has influence over its students’ lives. In schools with high value-added scores, young people are attending and attaining more than would be expected. Where a school is perceived to be meaningful by its students, this may be associated with changes in outcomes that can be assessed through objective attendance and attainment measures within the value-added scale.

Whether a school is perceived to be meaningful by its students will depend on the two cultural orders being transmitted by the school—the regulatory and instructional orders (Bernstein, 1975). The regulatory order defines the student behaviours that are acceptable to the school through its rules and values, while the instructional order sets the educational aspirations of the schools—both in terms of product of educational attainment and the means by which this achieved (Markham et al., 2021). When students are committed to the school orders that they will display high levels of school connectedness, which reflects a sense of belonging and purpose in school, this will outwardly result in greater attendance and attainment outcomes. Aveyard et al., (2004) found evidence that the value-added measure had face and concurrent validity as a means of measuring transmission of the instructional and regulatory orders.

When young people transition from primary to secondary school, it is the point at which the young person’s own cultural background first interacts with the culture of the secondary school. This interaction may be positive, where the two cultures align and complement each other, or more problematic where the young person finds the school culture to be incongruent with their home values and aspirations. Both social class and cultural heritage can determine the values, aspirations and expectations that a young person will bring into the school environment with them. However, as schools typically reflect a middle class understanding of the aims and means of education, often focused on material prosperity (Markham et al., 2021), young people who are not from middle class backgrounds may find these aims and means incongruent with their own value systems.

For a young person who finds the school culture to be opposed to or to conflict with their home culture, transition may be a more stressful experience. It is therefore unsurprising that transition is a point in the life course when some students begin to disengage from education (Hascher & Hadjar, 2018) and mental health inequalities are at risk of widening (Moore et al., 2020). Schools that are highly meaningful may make transition less problematic for young people by being more culturally aligned and relatable for students, requiring fewer personal resources to successfully navigate the transition process. Low school-level meaningfulness, however, may be reflected in high levels of frustration in students, a risk factor for school disconnectedness (Markham et al., 2021).

This value-added ‘meaningfulness’ measure has been used to explain why smoking uptake, early alcohol initiation, heavy alcohol consumption, illicit drug use, stealing and fighting are lower in some schools than others (Aveyard et al., 2004; Bisset et al., 2007; Bonell et al., 2017; Markham et al., 2008; Tobler et al., 2011). These findings provide support for the idea that students connected to the two school orders are less likely to reject valued school identities and seek affiliation with subcultures where deviant behaviour is more likely to be promoted, as argued by Markham (2008, 2015). While the measure has not been used to explain differences in mental health and wellbeing outcomes between schools, a similar logic applies—young people who perceive the school to be ‘meaningful’ are likely to be more connected to the school orders and feel a greater sense of belonging and self-esteem, have more peer support within school and feel more able to seek support from teachers when stressed or worried, resulting in better mental health outcomes.

### Subjective Measures of School Environment

Alongside objective approaches, self-report measures that seek to understand young people’s own perceptions of the school environment can help to provide subjective measures of the extent to which a young person may be committed to school orders. In particular, self-report measures of school connectedness provide important insights into the school environment. While there is no globally accepted definition of school connectedness, it is typically treated as a multi-dimensional construct that measures a number of different dimensions including acceptance and care, belonging, liking and enjoying, respect and support (García-Moya et al., 2019). A meta-analysis of the relationship between school connectedness (including measures of social affiliation, school belonging and attitudes about school importance) and health risks found that self-reported school connectedness had an important protective effect on violent behaviours, high-risk substance abuse, risky sexual behaviours and mental health (including anxiety and depression) outcomes across all stages of school (elementary, middle and high school) (Rose et al., 2024). Furthermore, there is evidence of a longitudinal effect, with school connectedness as a significant protective factor for future depression and anxiety (Raniti et al., 2022). A positive relationship between school connectedness and psychological wellbeing has also been demonstrated through meta-analysis (Yuen & Wu, 2024).

Previous analysis has shown that measures of school connectedness are significantly associated with mental health difficulties in the first year after transition to secondary school (Donaldson et al., 2024), and that feeling connected to school and having peer support across transition is protective of mental health (assessed using items related to depression, anxiety and stress) (Lester & Cross, 2015). However, there has not been any research to explore how measures of school connectedness might fit alongside a school-level measure of meaningfulness.

This research will use primary and secondary school linked data from Wales, UK, to follow young people across school transition and consider how factors on both sides of transition might be associated with post-transition mental health and wellbeing outcomes. The three research questions are therefore:

1. Are individual-level factors (pre-transition mental health difficulties, SES, gender and being worried about transition) associated with mental health difficulties and mental wellbeing outcomes following transition to secondary school?
2. Are young people’s perceptions of secondary school (sense of belonging, whether teachers care about them and whether their ideas are taken seriously) associated with mental health difficulties and mental wellbeing outcomes following transition to secondary school?
3. To what extent is meaningfulness, as a measure of secondary school environment, associated with mental health difficulties and mental wellbeing outcomes following the transition to secondary school?

## Methods

This analysis uses linked data from 506 students across two school surveys in Wales, UK. The first (‘CHETS’) took place when students were in the final year of primary school (year 6, aged 10–11 years) and the second, the School Health Research Network (SHRN) survey, took place the following academic year in the first year of secondary school (year 7, age 11–12 years). CHETS was carried out in the spring/summer of 2019 prior to transition and SHRN in the autumn/winter of 2019, post-transition.

Both surveys involved three levels of informed consent. Schools signed an agreement to take part in a survey and provided parents with study information and details of opt-out consent procedures, provided by the research team. Parents were provided materials two weeks before the survey and asked to notify the school if they did not want their child to take part. For the CHETS survey in primary schools, researchers visited each school and provided pupils with an oral description of the study within their usual classroom setting. Pupils then completed an assent form prior to participation in the survey, conducted using paper forms. For the SHRN survey in secondary schools, the survey was conducted online in schools. In both surveys, pupils were advised that participation was voluntary, that they could choose not to complete any items they did not want to and that they could stop at any time without giving a reason. Ethical approval for both surveys, as well as the secondary data analysis provided in this paper, were provided by the Cardiff University School of Social Sciences Research Ethics Committee (SREC/2700, SREC/3251 and SREC/3652).

The full CHETS survey included 2170 respondents from 73 primary schools across Wales (Moore et al., 2020). The full SHRN survey included 119,388 respondents in years 7 to 11 from 198 secondary schools across Wales (Page et al., 2021). Within CHETS, pupils were asked to provide their name, month and year of birth and the secondary school they anticipated transitioning to, in order to enable linkage across the transition. In the SHRN survey, pupils were asked to provide their name, date of birth and to indicate which primary school they attended. This linkage information was requested at the end of the SHRN survey. Approximately 19% of Year 7 pupils in Wales did not complete the SHRN survey. Of those who did, approximately 15% did not reach the end of the survey, while pilot work indicated that of those who reached the end of the survey, around half would provide accurate data for linkage (Morgan et al., 2020). Hence, it was anticipated that approximately 30% of CHETS participants would be linked to SHRN survey responses.

Of the students for whom data was linked, the majority transitioned with a large group of their peers to the same secondary school. Ninety students were excluded from analyses due to non-standard transition. These included situations where young people from an English language medium primary transitioned to a Welsh medium secondary, while the majority of their cohort transitioned to a common English language secondary; where young people transitioned to a faith school rather than their catchment secondary and in one case where young people were split at transition into separate boys and girls secondary schools. Mapping and understanding these different types of non-standard transition, where young people’s experiences are likely to be different to those transitioning with the majority of their cohort, would require a larger sample size. Given the relatively small sample, it was therefore decided to focus on the more dominant, standard form of primary-to-secondary transition. A flowchart of how the final sample of 506 students was obtained is given in Online Appendix 1. The pupils included in the final sample attended 59 different primary schools and transitioned to 51 different secondary schools in Wales. Primary schools were all nested within secondary schools, that is, all students within a given primary school transitioned to the same secondary school.

The characteristics of the final sample of 506 young people were compared to the year 7 students included in the full SHRN survey (*n* = 26,786) (Appendix 2) to check for representativeness. The sample included in this paper had a higher percentage of girls than the nationally representative SHRN survey (54.0% vs. 49.8%) and a lower percentage of young people who live with both parents (65.9% vs. 69.7%). However, ethnicity and family affluence were closely aligned as were the outcome measures and school connectedness covariates.

### Measures

#### School-Level Meaningfulness Measure

A value-added meaningfulness measure was developed in line with approaches used in previous studies (Aveyard et al., 2004; Bisset et al., 2007; Bonell et al., 2017; Markham et al., 2008; Tobler et al., 2011), using data from Stats Wales via the ‘my local school’ website, use of which is licenced under the Open Government Licence v3.0 (Welsh Government, 2022). As of January 2021, there were 205 state secondary schools in Wales. Of these, six had opened in the previous five years and so were yet to have any attainment data. The remaining 199 secondary schools were included in the analysis.

Variables were selected to align as closely as possible to previous meaningfulness scales, but were restricted to what data was publicly available. To create the meaningfulness measure, data were extracted on attainment and attendance, and on five predictor variables: the three-year average percentage of students eligible for FSMs; the welsh index of multiple deprivation (WIMD) score for each school’s postcode; the proportion of females in the school; the proportion of students without White British ethnicity; and the proportion of young people with special educational needs (SEN). Attainment was measured using the Capped 9 Points Score, which is calculated for each student in year 11 (age 16) in Wales based on achievement at GCSE level (Welsh Government, 2019). For attendance, the Welsh Government provides a percentage of half days attended by students across the school year. This was averaged for 2015–2019 to provide a five-year average for each secondary school. Achievement and attendance were positively correlated (r* =* 0.74).

Two linear regression models were developed (Tobler et al., 2011), one with the Capped points 9 score as an outcome, and the other with the attendance measure as an outcome. All predictor variables were added to both models. Residuals from each model were stored and standardised. The residuals represent the difference between expected and actual scores for each school and outcome, given the school’s demographic composition. Standardised residuals for achievement and attainment were moderately correlated in the two models (*r* = 0.41), suggesting that schools with higher attainment also tend to have higher levels of attendance. A principle component analysis (PCA) was run for the residuals from the attendance and attainment models. One component was retained, accounting for 70.41% of variance and with loadings of + 0.71 for both attendance and attainment residuals. The residuals were summed and standardised to have a mean of zero and a standard deviation of one. The meaningfulness scores were merged into the student survey dataset.

#### Mental Health Measures

Outcome measures were selected based on their validation for different age groups. The strengths and difficulties questionnaire (SDQ) and short Warwick Edinburgh mental wellbeing scale (SWEMWBS) are only validated for use from age 11 (Goodman, 1997; Melendez-Torres et al., 2019; Stewart-Brown et al., 2009) and could therefore not be used in the primary school survey. The me and my feelings (M&MF) questionnaire was selected for use in year 6 as is validated for use from age eight (Deighton et al., 2013). All items across the mental health scales are listed in Appendix 3.

M&MF contains 16 items, of which 10 comprise an emotional difficulties scale and six comprise a behavioural difficulties scale. Respondents respond ‘never’, ‘sometimes’ or ‘always’ to a list of items. For the emotional difficulties scale, items include: ‘I feel lonely’, ‘I cry a lot’, ‘Nobody likes me’, ‘I have problems sleeping’. For the behavioural difficulties scale, items include: ‘I get very angry’, ‘I lose my temper’, ‘I do things to hurt people’, ‘I break things on purpose’. Each item is scored from 0 to 2 and summed. Cronbach’s α was 0.78 for emotional difficulties and 0.77 for behavioural difficulties.

The SWEWMBS asks respondents to the state the extent to which seven statements describe their experience over the last two weeks. Responses scored from 1 to 5 on a Likert scale, and higher scores indicating higher wellbeing (*α* = 0.79). Items include: ‘I’ve been feeling optimistic about the future’, ‘I’ve been feeling useful’, ‘I’ve been feeling relaxed’, ‘I’ve been dealing with problems well’, ‘I’ve been thinking clearly’, ‘I’ve been feeling close to other people’ and ‘I’ve been able to make up my own mind about things’.

The self-report SDQ measure is a 25-item scale comprising five subscales of five items, with each item scored from 0 to 2 (‘Not true’, ‘somewhat true’, ‘certainly true’). One of the subscales (the prosocial scale) was not used in this analysis as it represents a narrower conceptualisation of strengths than the SWEMWBS measure and does not contribute to the scoring of difficulties. Young people are asked to answer based on their experiences over the past six months. Four of the five subscales can be summed to give a total difficulties score (*α* = 0.83): emotional problems (*α* = 0.73), conduct problems (*α* = 0.61), hyperactivity (*α* = 0.76) and peer problem subscales (*α* = 0.48). These alpha values were mostly in line with the full nationally representative SHRN survey, although the peer problems value was lower in this sample (Online Appendix 2). They are also in line with Goodman (2001)’s original paper which found that despite not all scales having high alpha values, they remained useful for mental health screening purposes. Questions for emotional problems include: ‘I get a lot of headaches’, ‘I worry a lot’, ‘I am nervous in new situations. I easily lose confidence’. Conduct problems items include: ‘I get very angry and often lose my temper’, ‘I usually do what I am told’ (reverse-scored), ‘I fight a lot. I can make other people do what I want.’ Hyperactivity items include: ‘I am restless, I cannot stay still for long’, ‘I am constantly fidgeting or squirming, ‘I am easily distracted, I find it difficult to concentrate’. Peer problem items include: ‘I am usually on my own. I generally play alone or keep to myself’, ‘Other people my age generally like me’ (reverse-scored), ‘Other children or young people pick on me or bully me’.

As emotional problems and conduct problems are not necessarily present concurrently in the same young people, and the M&MF scale includes emotional and behavioural difficulties scales that aligned with these two subscales, the analysis also used the two SDQ subscales (emotional problems and conduct problems) separately as dependent variables, with their associated M&MF subscale as a covariate.

#### School Connectedness Measures

Three items were used to assess aspects of school connectedness, with young people asked to rate on a five-point Likert scale the extent to which they agreed or disagreed that ‘I feel like I belong at this school’, ‘I feel that my teachers care about me as a person’ and ‘At our school my ideas are taken seriously’. Items were reverse-scored so that higher values indicated more positive school connectedness. The latter two items are derived from the World Health Organization (WHO) Health Behaviour in School-aged Children (HBSC) survey (Badura et al., 2024).

#### Worried About Transition

A single item from the year-6 survey that asked students to what extent they were worried about the transition to secondary school was used. It was scored on a Likert scale from 1 (not at all) to 5 (very much).

#### Demographic Measures

The family affluence scale (FAS) was administered to assess SES of participants in year 7. The six items include: whether the family owns a car or other vehicle, whether the young person has their own bedroom, the number of computers in the home, how many bathrooms are in the home, the number of holidays in the past year and whether they have a dishwasher (Boyce et al., 2006). It is scored out of 13. The proportion of students eligible for free school meals within each school was used as a measure of school affluence based on the three-year average (from 2017 to 2019). To provide a measure of gender, young people were asked ‘Are you male or female?’ and answer options were ‘Male (a boy)’, ‘Female (a girl)’, ‘Neither word describes me’, ‘I do not want to answer’.

#### Missing Data

For measures made of multiple items, where more than half of the items had a response, a pro-rated score was calculated. There was an overall relatively low level of missing data across the individual- and school-level variables within the final sample of 506 young people (Online Appendix 4). In order to account for missingness, data were cleaned in Stata 15.0 and then transferred to R version 4.3.1 for multiple imputation (MI). Substantive model compatible multi-level MI was used (Quartagno & Carpenter, 2022; RDocumentation, 2023). Four separate imputation datasets were created (one for each outcome variable). In addition to including all outcome and predictor variables within every imputation, a number of additional auxiliary variables were selected to support imputation (Online Appendix 4). In all imputations, all four SDQ subscales and the total difficulties score were included, and the six family affluence scale items were included separately alongside the overall score. Burn-in and between-imputation updates were set to 1000, and five imputations carried out per substantive model. Descriptive statistics of imputed outcome variables are provided in Appendix 5. Imputation resulted in a small percentage of values that exceeded the minimum and maximum bounds of the continuous outcome variables; however, simulations suggest that unbiased model estimates are obtained by retaining the imputations as received rather than imposing restrictions on range either during or after multiple imputation (Rodwell et al., 2014). A sensitivity analysis (Appendices 7–9) confirmed no difference with the main model. Imputed datasets were transferred back into Stata for multi-level analysis.

#### Multi-Level Modelling

A series of hierarchical linear mixed-effects models for each outcome measure were developed to answer the research questions. A likelihood ratio (LR) test was run on the first imputation (it is not possible to run LR tests on imputed data) to determine whether a three-level model (students in primary schools in secondary schools) was a better fit for the data than a two-level model (students in secondary schools). For all outcomes, the three-level model was not a significantly better fit and therefore two-level models (students in secondary schools) were run for all outcomes. Unadjusted models were run to calculate intra-class correlation coefficients for secondary school mental health outcomes (ICCs) (Appendix 10). The first adjusted model included pre-transition mental health measures, gender and family affluence. The SDQ emotional and conduct problems subscales were matched with their relevant pre-transition indicator (the former with the pre-transition emotional difficulties variable, and the latter with the pre-transition behavioural difficulties variable). For SWEMWBS and SDQ total difficulties, both pre-transition mental health measures were controlled for. The second adjusted model included all remaining individual-level predictors (being worried about transition and the school connectedness items). The final model added the school-level variables. All imputed models and sensitivity analyses are presented in Appendices 6–9.

## Results

### Descriptive Statistics

The standardised meaningfulness scores produced for each school were normally distributed. In the full meaningfulness dataset (total secondary schools = 199), there were 25 ‘high’ meaningfulness schools (> + 1SD from the mean) and 27 ‘low’ meaningfulness schools (< -1SD from the mean). Meaningfulness had little correlation with the percentage of students eligible for free school meals (*r* = 0.003), but greater correlation with attainment (*r* = 0.58) and attendance (*r* = 0.44).

Within the CHETS-SHRN school data, over half of pupils were female (54%) and the majority (84%) were of White British ethnicity. Sample descriptives are presented in Table 1. M&MF and SDQ scales were moderately negatively correlated with SWEMWBS (Table 2).

**Table 1:** Descriptive statistics of sample

	*N* (%)
*Gender*	
Male	233 (46.0%)
Female	273 (54.0%)
*Ethnicity*	
White British	413 (83.8%)
Other Ethnicity	80 (16.2%)
*Family structure*	
Live with Mum and Dad	328 (65.9%)
Other family structure	170 (34.1%)
*Family affluence*	
Low (0–8)	170 (35.3%)
Medium (9–10)	144 (29.9%)
High (11–13)	167 (34.7%)

	Mean (standard deviation) range
Pre-transition emotional difficulties (M&MF)	6.2 (3.6) 0–19
Pre-transition behavioural difficulties (M&MF)	2.7 (2.3) 0–11
Post-transition total difficulties (SDQ)	10.7 (6.4) 0–31
Post-transition emotional problems (SDQ)	3.4 (3.4) 0–10
Post-transition conduct problems (SDQ)	1.8 (1.8) 0–8
Post-transition mental wellbeing (SWEMWBS)	22.6 (4.6) 7–35
Worried about secondary	2.8 (1.4) 1–5
School belonging	4.1 (0.9) 1–5
Teachers care	4.0 (1.0) 1–5
My ideas taken seriously	3.7 (1.1) 1–5

SWEMWBS, short Warwick Edinburgh mental wellbeing scale; SDQ, strengths and difficulties questionnaire; M&MF, me and my feelings questionnaire. Statistics calculated pre−multiple imputation

**Table 2:** Correlation (Pearson’s r) between mental health and wellbeing scales

	Pre-transition emotional difficulties (M&MF)	Pre-transition behavioural difficulties (M&MF)	Post-transition total difficulties (SDQ)	Post-transition emotional problems (SDQ subscale)	Post-transition conduct problems (SDQ subscale)	Post-transition mental wellbeing (SWEMWBS)
Pre-transition emotional difficulties (M&MF)	1.00	–	–	–	–	–
Pre-transition behavioural difficulties (M&MF)	0.34 (*p* < 0.001)	1.00	–	–	–	–
Post-transition total difficulties (SDQ)	0.39 (*p* < 0.001)	0.29 (*p* < 0.001)	1.00	–	–	–
Post-transition emotional problems (SDQ subscale)	0.43 (*p* < 0.001)	0.15 (*p* < 0.001)	0.77 (*p* < 0.001)	1.00	–	–
Post-transition conduct problems (SDQ subscale)	0.23 (*p* < 0.001)	0.38 (*p* < 0.001)	0.74 (*p* < 0.001)	0.33 (*p* < 0.001)	1.00	–
Post-transition mental wellbeing (SWEMWBS)	− 0.31 (*p* < 0.001)	− 0.25 (*p* < 0.001)	− 0.57 (*p* < 0.001)	− 0.47 (*p* < 0.001)	− 0.39 (*p* < 0.001)	1.00

SWEMWBS, short Warwick Edinburgh mental wellbeing scale; SDQ, strengths and difficulties questionnaire; M&MF, me and my feelings questionnaire. Correlations calculated pre−multiple imputation

### Multi-Level Analysis

Unadjusted ICCs ranged from 0.024 for emotional difficulties to 0.067 for conduct problems, in line with other research on mental health in schools (Parker et al., 2023; Shackleton et al., 2016) (Appendix 10). Table 3 presents the final model for each outcome variable.

**Table 3:** Final multi-level regression models for all outcomes (*n* = 506)

	Post-transition mental wellbeing (SWEMWBS)		Post-transition total difficulties (SDQ)		Post-transition emotional problems (SDQ subscale)		Post-transition conduct problems (SDQ subscale)	
	*β* (95%CI)	*P*-value	*β* (95%CI)	*P*-value	*β* (95%CI)	*P*-value	*β* (95%CI)	*P*-value
Emotional difficulties (M&MF)	− 0.12 (− 0.24, − 0.01)	*p* = 0.030	0.43 (0.28, 0.59)	*p* < 0.001	0.20 (0.14, 0.26)	*p* < 0.001	–	–
Behavioural difficulties (M&MF)	− 0.30 (− 0.46, − 0.13)	*p* < 0.001	0.40 (0.13, 0.66)	*p* = 0.004	–	–	0.28 (0.21, 0.35) *p* < 0.001	*p* < 0.001
Female	− 0.01 (− 0.72, 0.69)	*p* = 0.975	− 0.87 (− 1.82, 0.08)	*p* = 0.072	0.35 (− 0.04, 0.75)	*p* = 0.078	− 0.39 (− 0.68, − 0.09)	*p* = 0.010
Family affluence scale	0.18 (0.03, 0.34)	*p* = 0.022	− 0.30 (− 0.50, − 0.09)	*p* = 0.005	− 0.08 (− 0.16, 0.01)	*p* = 0.079	− 0.05 (− 0.12, 0.02)	*p* = 0.133
Worried secondary	− 0.28 (− 0.57, 0.01)	*p* = 0.055	0.21 (− 0.19, 0.61)	*p* = 0.300	0.23 (0.02, 0.43)	*p* = 0.031	0.04 (− 0.07, 0.15)	*p* = 0.499
School belonging	0.82 (0.39, 1.25)	*p* < 0.001	− 0.74 (− 1.32, − 0.15)	*p* = 0.013	− 0.31 (− 0.57, − 0.06)	*p* = 0.016	− 0.12 (− 0.32, 0.09)	*p* = 0.254
Teacher cares	0.79 (0.38, 1.20)	*p* < 0.001	− 1.71 (− 2.31, − 1.12)	*p* < 0.001	− 0.20 (− 0.45, 0.05)	*p* = 0.109	− 0.42 (− 0.62, − 0.21)	*p* < 0.001
Ideas taken seriously	0.89 (0.51, 1.27)	*p* < 0.001	− 0.97 (− 1.45, − 0.48)	*p* < 0.001	− 0.47 (− 0.68, − 0.26)	*p* < 0.001	− 0.07 (− 0.23, 0.10)	*p* = 0.424
School % free school meals	− 0.00 (− 0.07, 0.07)	*p* = 0.989	− 0.05 (− 0.15, 0.06)	*p* = 0.403	− 0.03 (− 0.07, 0.00)	*p* = 0.079	− 0.01 (− 0.04, 0.02)	*p* = 0.620
Meaningfulness	0.54 (0.05, 1.03)	*p* = 0.030	− 0.00 (− 0.73, 0.72)	*p* = 0.993	0.07 (− 0.18, 0.32)	*p* = 0.594	− 0.05 (− 0.29, 0.18)	*p* = 0.655
Residual variance	13.99 (12.28, 15.93)		25.61 (22.40, 29.27)		4.46 (3.91, 5.09)		2.44 (2.14, 2.78)	

SWEMWBS, short Warwick Edinburgh mental wellbeing scale; SDQ, strengths and difficulties questionnaire; M&MF, me and my feelings questionnaire (pre−transition measure)

For all outcomes, pre-transition mental health was a significant predictor of post-transition mental health. Post-transition conduct problems were significantly lower for females [*β* = − 0.39 (− 0.68, − 0.09)] than for males. Higher family affluence was significantly associated with lower post-transition total difficulties [*β* = − 0.30 (− 0.50, − 0.09)] and higher post-transition mental wellbeing [*β* = 0.18 (0.03, 0.34)]. Young people who were worried about transition in year 6 were significantly more likely to have emotional difficulties in year 7 [*β* = 0.23 (0.02, 0.43)].

Higher perceptions of school connectedness post-transition were associated with higher post-transition wellbeing and lower post-transition difficulties, although the strength of the association varied by school connectedness item and outcome measure. Sense of belonging and having their ideas taken seriously were associated with significantly higher wellbeing, lower total difficulties and lower emotional difficulties. Perceptions that their teachers care them were associated with significantly higher mental wellbeing, lower total difficulties and lower conduct problems.

At the school level, % of students eligible for free school meals within each secondary school was not a significant predictor in any of the models. High school meaningfulness in secondary school was significantly associated with more positive post-transition mental wellbeing outcomes [*β* = 0.54 (0.05, 1.03)]. There was no evidence of a relationship between meaningfulness and the post-transition mental health difficulties outcomes.

## Discussion

This analysis has sought to investigate both individual-level and school-level predictors of mental health in the transition from primary to secondary school. For all outcome measures and models, pre-transition scores of emotional and/or behavioural difficulties were significant, suggesting that the most consistent predictor of mental health difficulties and low mental wellbeing in year 7 is having equivalent difficulties in year 6. It highlights the importance of early intervention for young people’s mental health. Recent research using data from a 2022–2023 survey of young people in Wales (*n* = 32,606) found that 24% already had elevated or clinically significant emotional difficulties in year 3 (age 7–8), three years before transition to secondary school (Donaldson et al., 2023b).

At the individual level, being worried about the transition to secondary school was associated with higher emotional problems in year 7, even after controlling for related difficulties in year 6 or family affluence. This chimes with the previous research (e.g. Jindal-Snape et al., 2020; Moore et al., 2021; Rice et al., 2021) but also raises the question of whether young people who worry about transition may be alike in ways not considered within this analysis, for example, having additional learning needs (ALN). Research has shown that young people with ALN are particularly vulnerable across the transition period and may require targeted intervention to address anxiety (Neal et al., 2016).

Family affluence was a significant predictor of total difficulties and mental wellbeing. The relationship between mental illness and SES has been extensively explored (e.g. Reiss, 2013), but findings for mental wellbeing tend to be more nuanced. While there appears to be a clear association between low wellbeing and low SES, there is less evidence of an association between high wellbeing and SES (Nielsen et al., 2016; Santini et al., 2020; Stewart-Brown et al., 2015). There is some research that suggests that modifiable factors such as spending time with family and friends, volunteering, engaging in a challenging activity or hobby may be more important mechanisms for building high mental wellbeing than focusing on SES (Santini et al., 2020). This research, however, found that higher SES was protective across both mental health difficulties and mental wellbeing outcomes.

Higher perceptions of the three school connectedness items were associated with fewer mental health difficulties and higher mental wellbeing. It highlights the importance of young people having a strong sense of belonging at school, feeling that their teachers care about them and that their ideas are taken seriously. This reflects previous research that points towards a protective effect of school connectedness on mental health (Raniti et al., 2022; Rose et al., 2024; Yuen & Wu, 2024) and that school-based interventions to improve young people’s mental health may operate via improvements in school connectedness (Melendez-Torres et al., 2021). It is also possible that there is a bidirectional effect at play, where young people with better mental health feel more able to connect to their school environment and build a sense of belonging. Future research should consider whether there are some groups of young people who particularly struggle to build school connectedness, perhaps due to mental health difficulties or ALN. For example, there is longitudinal evidence that high depressive symptoms are associated with lower school connectedness six months later, which the authors suggest may be due to depressive symptoms resulting in a lack of motivation for studying and participating in other school activities, problems with memory and attention, and higher school absence (Klinck et al., 2020). Hebron (2018) also found that young people with autism had lower level of school connectedness across the primary to secondary school transition than their typically developing peers, but the reasons for this were unclear. Research should consider how targeted support can be provided to ensure young people with specific support needs are given every opportunity to build new relationships with peers and teachers, and to build a sense of belonging as they begin their new school.

For mental health difficulties, there were some differences in which individual-level variables had significant associations with the total difficulties scale and the two SDQ subscales. Being female was associated with lower conduct problems, but not emotional problems or total difficulties. Family affluence was associated with the total difficulties scale, but not the two subscales. Being worried about secondary school was associated with emotional problems, but not total difficulties or conduct problems. Finally, of the school connectedness items, both belonging and having their ideas taken seriously were only associated with total difficulties and emotional problems; perceptions of teacher care was associated with total difficulties and conduct problems but not emotional problems. These findings highlight the nuance of how different aspects of mental health might be associated with different individual-level predictors, and while some of these associations are relatively well documented—e.g. higher externalising problems in boys than girls (Leadbeater et al., 1999)—more research to understand these relationships, particularly around school connectedness is warranted.

School meaningfulness was associated with mental wellbeing, but not mental health difficulties. There is strong evidence that the two outcomes frequently have different predictors. For example, in migrants to Australia, younger age and being a student was predictive of mental distress but not mental wellbeing, while being self-employed and identifying with Australia was associated with mental wellbeing but not distress (du Plooy et al., 2019). Huppert & Whittington (2003) found that employment had a greater impact on mental wellbeing than mental illness, but poor physical health and a lack of social support had a bigger negative impact on mental illness and less effect on mental wellbeing. In a large sample of British adults, many demographic characteristics (age, gender, ethnicity, employment, financial strain, poor housing and household composition) were differentially associated with symptoms of mental illness and mental wellbeing (Hu et al., 2007).

This has implications for intervention development, as it should not be assumed that the same theory of change will apply to efforts to improve both mental illness and mental wellbeing. The effect of school-level meaningfulness on mental wellbeing was small but important. After controlling for individual-level covariates and school-level FSM, a one-unit increase in school meaningfulness will result in an average 0.54-unit increase in mental wellbeing. A one-unit increase in meaningfulness would be slightly more than one standard deviation within this sample (sd = 0.85) and may seem like an unobtainable shift for many schools. However, even a much smaller increase in meaningfulness could have a big impact due to the large numbers of young people within each school (Rose, 2001). The relationship found here between mental wellbeing and meaningfulness suggests that the latter may be particularly associated with the assets associated with good mental wellbeing, such as autonomy, positive relationships, purpose in life, environmental mastery, self-acceptance and personal growth (Ryff, 2013) rather than the deficits associated with mental illness. By increasing school meaningfulness, the school environment is aligned with young people’s own purpose in life and personal growth in particular.

However, the direction of effect between meaningfulness and mental wellbeing should also be considered. While a high meaningfulness school-culture may result in higher mental wellbeing in students, the opposite effect may also be true: a school with particularly high numbers of students with high wellbeing would be likely to have higher attendance and attainment leading to a higher meaningfulness score. Further analysis using structural equation modelling to fully understand the pathways between the measures of attendance, attainment, meaningfulness and mental wellbeing might help to better understand the relationships between each of them.

More research is also needed into the practical steps that schools can make to increase their meaningfulness as conceptualised here. A school that is highly meaningful for one community may not be for another as it emerges through the interaction between individual students with their own social and cultural heritage, and the culture of the school as determined by school organisation, curriculum and pedagogic practice (Markham & Aveyard, 2003). Schools need to understand the communities that they serve, seek to widen their range of valued school identities and coproduce their culture with their student body. Exploring the lived experiences of young people experiencing the transition from primary to secondary school would help better understand the mechanisms by which students internalise school culture, produce perceptions about school’s regulatory and instructional orders, the behavioural and affective responses that follow, and how students use agency to determine these responses. Greater understanding of this process would support development of interventions to support students over the transition period.

Situating the findings within the Welsh context also offers opportunities for supporting young people’s mental health. In 2021, the Welsh Government published a framework for embedding a whole school approach to emotional and mental wellbeing (Welsh Government, 2021). Key to the approach is that mental health of both students and staff needs to be considered at every level of the school environment, from curriculum, leadership, the physical school environment and transition points. The concepts of meaningfulness and school connectedness therefore resonate strongly with this approach and highlight the importance of listening to the needs of students, teachers and parents, and in doing so centring belonging, efficacy and voice to create a supportive, mental health-promoting environment.

This study has a number of key strengths. This is the first analysis of a proxy value-added educational measure as a means of understanding the relationship between school-level meaningfulness and young people’s mental health. It offers an important insight into young people’s experiences over the primary to secondary school transition, highlighting how individual factors, as well as school environment might influence young people’s mental health during an important educational life event.

There are also a number of potential limitations to this analysis that should be highlighted. Unlike some of the other studies where a similar meaningfulness measure was developed (Aveyard et al., 2004; Bisset et al., 2007; Bonell et al., 2017; Markham et al., 2008; Tobler et al., 2011), it was not possible to include a measure of the proportion of students speaking English as an additional language within the regression model due to missing data, and the WIMD variable was based on the deprivation score associated with the school postcode which may not fully represent the intake to the school. The calculation for producing the Capped 9 Points attainment scale changed in 2019, making comparison with previous years problematic. Therefore, only data from 2019 was used in the analysis as it was not possible to create a five-year average as in previous analyses. It was not feasible to combine with 2020 or 2021 data due to the impact of the coronavirus pandemic on data availability and exam results. The individual-level school connectedness items were also limited to those available within the secondary data, and future research might wish to consider using other validated scales.

A number of hypothesised relationships were non-significant in this analysis when considered based on *p*-values alone. However, *p*-values have clear limitations and their role in determining whether an effect is important or likely to be due to chance is not absolute, particularly in a sample that may be underpowered. Increasingly researchers are arguing for a more nuanced depiction of findings (Halsey, 2019; Ho et al., 2019). In this analysis, where *p*-values were in the expected direction and only marginally insignificant, increasing sample size (and therefore power) might find significant effects (Carnahan & Brown, 2024). Taking non-significance as evidence of a null effect may result in potential intervention strategies or policy recommendations being overlooked. Equally, interpreting a non-significant trend as significant may also result in incorrect assumptions about what might work to improve young people’s mental health. Where findings are only marginally non-significant, further research should be undertaken to better understand the relationship between variables.

It is also possible that unaccounted for student or school-level effects may be confounding the relationship between meaningfulness and mental wellbeing. As argued by Aveyard et al., (2004), parental attitudes in particular might result in a young person being sent to a school that reflects a family’s values—i.e. some parents might deliberately choose schools that are highly meaningful. The value-added score is not available to parents; however, both attainment and attendance data are available, and there were moderate correlations between both of these indicators and meaningfulness, which might have some influence over choice of school. Choice of school does not just mean at the point of application, but also when parents consider where to live and which school catchment area they are in. Although young people in this study attended secondary schools with the majority of their cohort, the decision to send them to that particular school was likely made many years before, particularly in more affluent areas with the highest performing schools. It may also be that local residents have some information about school culture based on word of mouth and personal experience, and future research could consider whether parental perceptions of school culture reflect actual meaningfulness scores.

The sample may not be fully representative of the Welsh school population. While both surveys used were representative in their entirety, only a subsample of young people who could be linked across the primary to secondary school transition were included in this analysis. The sample had a higher proportion of girls and a lower proportion of young people living with both parents than in the full SHRN survey; however, other variables were closely matched. There may also be confounding variables not measured or included in the analysis that might influence the findings.

## Conclusion

This research highlights the importance of individual- and school-level factors in understanding young people’s experiences of school transition. It highlights opportunities for intervention to support young people across the transition process by targeting school-level factors, as well as underscoring the interplay between individual characteristics and school environment in mental health outcomes.

## Supplementary Information

Below is the link to the electronic supplementary material.

Supplementary file1 (DOCX 70 kb)

## Declarations

### Data access

Data are available on reasonable request from the School Health Research Network (shrn@cardiff.ac.uk).
