# Supplementary material for: Individual and School Environment Predictors of Mental Health and Wellbeing Across the Primary-to-Secondary School Transition
Source: School Ment Health. 2025 Jun 25;17(3):890–902. doi: 10.1007/s12310-025-09776-9 (PMC12479681; doi:10.1007/s12310-025-09776-9)
Supplement: Supplementary file 1 — Supplementary file1 (DOCX 70 kb) [file 12310_2025_9776_MOESM1_ESM.docx]

**Supplementary material**

CHETS data (n=2,170 year 6 students from 73 primary schools)

SHRN data (n=26,786

year 7 students from 198 secondary schools)

Total linked data (n=602 students)

Total after reduction for non-standard transitions (n=512 students)

60 primary schools;

52 secondary schools

Meaningfulness measure (n=199 secondary schools)

Of the 52 secondary schools, 51 had meaningfulness data

Final sample:

59 primary schools;

51 secondary schools; 506 students

**Appendix 1: Flowchart of data linkage and final sample size (n=506).**

**Appendix 2**

**Comparison of final sample characteristics (n=506) with year 7 students in the full SHRN survey (n=26,786)**

|  | Transition sample (n=506)  N (%) | Full SHRN sample (n=26,786)  N (%) |
| --- | --- | --- |
| Gender |  |  |
| Male | 233 (46.0%) | 13,066 (49.3%) |
| Female | 273 (54.0%) | 13,217 (49.8%) |
| Neither word describes me | 0 (0.0%) | 236 (0.9%) |
| Ethnicity |  |  |
| White British | 413 (83.8%) | 21,344 (84.3%) |
| Other Ethnicity | 80 (16.2%) | 3,967 (15.7%) |
| Family structure |  |  |
| Live with Mum and Dad | 328 (65.9%) | 14,736 (69.7%) |
| Other family structure | 170 (34.1%) | 6,399 (30.3%) |
| Family affluence |  |  |
| Low (0-8) | 170 (35.3%) | 8,980 (36.6%) |
| Medium (9-10) | 144 (29.9%) | 7,487 (30.5%) |
| High (11-13) | 167 (34.7%) | 8,053 (32.8%) |
|  |  |  |
|  | Mean (SD) Range | Mean (SD) Range |
|  |  |  |
| Post-transition total difficulties (SDQ) | 10.7 (6.4) 0-31 | 11.1 (6.7) 0-40 |
| Post-transition emotional problems (SDQ) | 3.4 (3.4) 0-10 | 3.3 (3.3) 0-10 |
| Post-transition conduct problems (SDQ) | 1.8 (1.8) 0-8 | 2.0 (1.9) 0-10 |
| Post-transition mental wellbeing (SWEMWBS) | 22.6 (4.6) 7-35 | 22.6 (4.9) 7-35 |
| School belonging | 4.1 (0.9) 1-5 | 4.0 (1.0) 1-5 |
| Teachers care | 4.0 (1.0) 1-5 | 4.0 (1.0) 1-5 |
| My ideas taken seriously | 3.7 (1.1) 1-5 | 3.6 (1.0) 1-5 |
|  |  |  |
|  | Cronbach’s α | Cronbach’s α |
|  |  |  |
| Post-transition emotional problems (SDQ) | 0.73 | 0.76 |
| Post-transition conduct problems (SDQ) | 0.61 | 0.60 |
| Post-transition hyperactivity (SDQ) | 0.76 | 0.72 |
| Post-transition peer problems (SDQ) | 0.48 | 0.56 |
| Post-transition total difficulties (SDQ) | 0.86 | 0.84 |
| Post-transition mental wellbeing (SWEMWBS) | 0.79 | 0.81 |

**Appendix 3: Items included in each mental health measure**

| **Me and My Feelings:**  **Emotional difficulties** | - ‘I feel lonely’ - ‘I cry a lot’ - ‘I am unhappy’ - ‘Nobody likes me’ - ‘I worry a lot’ - ‘I have problems sleeping’ - ‘I wake up in the night’ - ‘I am shy’ - ‘I feel scared’ - ‘I worry when I am at school’ | Responses:  0 = Never  1 = Sometimes  2 = Always |
| --- | --- | --- |
| **Me and My Feelings: Behavioural difficulties** | - I get very angry’ - ‘I lose my temper’ - ‘I hit out when I am angry’ - ‘I do things to hurt people’ - ‘I am calm’ (reversed scored) - ‘I break things on purpose’ | Responses:  0 = Never  1 = Sometimes  2 = Always |
| **Short Warwick Edinburgh Mental Wellbeing Scale** | - ‘I’ve been feeling optimistic about the future’ - ‘I’ve been feeling useful’ - ‘I’ve been feeling relaxed’ - ‘I’ve been dealing with problems well’ - ‘I’ve been thinking clearly’ - ‘I’ve been feeling close to other people’ - ‘I’ve been able to make up my own mind about things’ | Responses:  1 = Never  2 = Rarely  3 = Sometimes  4 = Often  5 = Always |
| **Strengths and Difficulties questionnaire: Emotional problems subscale** | - ‘I get a lot of headaches’ - ‘I worry a lot’ - ‘I am often unhappy, down-hearted or tearful’ - ‘I am nervous in new situations. I easily lose confidence’ - ‘I have many fears, I am easily scared’. | Responses:  0 = Not true  1 = Somewhat true  2 = Certainly true |
| **Strengths and Difficulties questionnaire:**  **Conduct problems subscale** | - ‘I get very angry and often lose my temper’ - ‘I usually do as I’m told’ - ‘I fight a lot. I can make other people do what I want’ - ‘I am often accused of lying or cheating’ - ‘I take things that are not mine from home, school or elsewhere’ | Responses:  0 = Not true  1 = Somewhat true  2 = Certainly true |
| **Strengths and Difficulties questionnaire:**  **Hyperactivity subscale** | - ‘I am restless, I cannot stay still for long’ - ‘I am constantly fidgeting or squirming’ - ‘I am easily distracted, I find it difficult to concentrate’ - ‘I think before I do things’ (reverse scored) - ‘I finish the work I am doing. My attention is good’ (reverse scored) | Responses:  0 = Not true  1 = Somewhat true  2 = Certainly true |
| **Strengths and Difficulties questionnaire:**  **Peer problems subscale** | - ‘I am usually on my own. I generally play alone or keep to myself’ - ‘I have one good friend or more’ (reverse scored) - ‘Other people my age generally like me’ (reverse scored) - ‘Other children or young people pick on me or bully me’ - ‘I get on better with adults than with people my age’ | Responses:  0 = Not true  1 = Somewhat true  2 = Certainly true |

| **Substantial model variables** | **Missingness** | **Odds of missingness predicted by substantive model and auxiliary variables? (p<0.05)** | **Variable predicted by auxiliary variables?** |
| --- | --- | --- | --- |
| SWEMWBS | 9/506 | No significant predictors | Higher life satisfaction (p<0.001); lower bully victimisation (p<0.001) |
| SDQ total difficulties | 38/506 | Completing survey in Welsh (p=0.024); being worried about transition (p=0.021); lower mental wellbeing (p=0.022); higher SDQ conduct scores (p=0.036); higher pre-transition behaviour difficulties (p=0.001); lower perceptions of teachers care (p=0.006). | Lower life satisfaction (p<0.001); higher bully victimisation (p<0.001) |
| SDQ emotional problems | 33/506 | Being worried about transition (p=0.013); lower mental wellbeing (p=0.045); higher pre-transition behaviour difficulties (p=0.010); lower perceptions of teachers care (p=0.002). | Lower life satisfaction (p<0.001); lower exercise (p=0.024); higher bully victimisation (p<0.001); ethnicity (p=0.018) |
| SDQ conduct problems | 30/506 | Being worried about transition (p=0.046); higher pre-transition behaviour difficulties (p=0.005); lower perceptions of teachers care (p=0.007). | Lower life satisfaction (p<0.001); higher bully victimisation (p<0.001); survey language (Welsh) (p=0.002) |
| M&MF emotional difficulties | 12/506 | No significant predictors | Lower life satisfaction (p<0.001); higher bully victimisation (p<0.001) |
| M&MF behavioural difficulties | 12/506 | Higher perceptions of teachers care (p=0.043). | Family structure (not living with both parents) (p=0.023); lower life satisfaction (p=0.002); higher bully victimisation (p=0.045) |
| Family affluence scale | 25/506 | Lower life satisfaction (p=0.005); higher bully victimisation (p=0.037); lower wellbeing (p=0.013); higher SDQ total difficulties (p=0.004); higher SDQ emotional difficulties (p=0.001); high SDQ peer problems (p=0.001); lower belonging (p=0.010); lower perceptions of ideas taken seriously (p=0.048). | Family structure (living with both parents) (p=0.021); higher life satisfaction (p=0.001); higher exercise (p<0.001); ethnicity (White ethnicity) (p=0.002) |
| Worried about transition | 10/506 | No significant predictors. | Lower life satisfaction (p=0.003); higher bully victimisation (p<0.001) |
| School belonging | 16/506 | Lower life satisfaction (p=0.009); higher SDQ total difficulties (p=0.043); lower perceptions of teachers care (p=0.001). | High life satisfaction (p<0.001); lower bully victimisation (p<0.001); ethnicity (not having white ethnicity) (p=0.002) |
| Teachers care | 14/506 | No significant predictors. | Higher life satisfaction (p<0.001); ethnicity (not having white ethnicity) (p=0.002) |
| Ideas taken seriously | 27/506 | Lower life satisfaction (p=0.003); completing survey in Welsh (p=0.042); lower wellbeing (p=0.027); higher SDQ conduct problems (p=0.015); lower perceptions of belonging (p=0.043). | Higher life satisfaction (p<0.001); lower bully victimisation (p<0.001); ethnicity (not having white ethnicity) (p=0.014) |
| Gender | 0/506 | N/A | N/A |
| Free school meal eligibility | 0/506 | N/A | N/A |
| Meaningfulness | 0/506 | N/A | N/A |

**Appendix 4: Predictors of missingness in substantive model variables and of the variables themselves using univariate multi-level analysis. Auxiliary variables included life satisfaction (students were asked to rate their life on a scale of 0-10, with 10 indicated as ‘I have the best possible life’ and 0 as ‘I have the worst possible life’. Scores were dichotomised so that 1 = scores of 8-10); ethnicity (for simplicity in this table, ethnicity has been binarized into White or other ethnicity, for the multiple imputation, a 12 category measure of ethnicity was used); exercise frequency (1 = exercise 4 or more times a week); whether a child has been a victim of bullying in the past few months at school (1 = yes); a language variable to determine whether the survey was carried out in Welsh or English; and family structure (live with mum and dad or other family structure). SWEMWBS = Short Warwick Edinburgh Mental Wellbeing Scale; SDQ = Strengths and Difficulties Questionnaire; M&MF = Me and My Feelings Questionnaire.**

| Outcome variable | Complete case | Imputation 1 | Imputation 2 | Imputation 3 | Imputation 4 | Imputation 5 |
| --- | --- | --- | --- | --- | --- | --- |
|  |  |  |  |  |  |  |
| SWEWMWBS | 22.60 (4.58)  [7.00, 35.00] | 22.54 (4.59)  [7.00, 35.00] | 22.58 (4.63)  [7.00, 35.00] | 22.56 (4.61)  [7.00, 35.00] | 22.57 (4.56)  [7.00, 35.00] | 22.58 (4.58)  [7.00, 35.00] |
| SDQ total difficulties | 10.66 (6.45)  [0.00, 31.00] | 10.81 (6.52)  [0.00, 31.00] | 10.87 (6.56)  [-0.62, 31.47] | 10.81 (6.50)  [-3.86, 31.00] | 10.75 (6.51)  [-2.74, 31.00] | 10.92 (6.53)  [0.00, 31.00] |
| SDQ emotional problems | 3.37 (2.55)  [0.00, 10.00] | 3.38 (2.54)  [-1.18, 10.00] | 3.37 (2.56)  [-0.95, 11.00] | 3.36 (2.54)  [-1.94, 10.00] | 3.39 (2.56)  [-0.91, 10.00] | 3.41 (2.54)  [-1.29, 10.00] |
| SDQ conduct problems | 1.84 (1.85)  [0.00, 8.00] | 1.88 (1.89)  [-0.70, 8.00] | 1.87 (1.86)  [-1.35, 8.00] | 1.86 (1.84)  [-1.24, 8.00] | 1.89 (1.88)  [-2.20, 8.00] | 1.88 (1.86)  [-0.89, 8.00] |

**Appendix 5: Comparison of mean (standard deviation) and [range] for the complete case analysis and each imputation. Statistics are from the imputed dataset aligned to each outcome variable. SWEMWBS = Short Warwick Edinburgh Menal Wellbeing Scale; SDQ = Strengths and Difficulties Questionnaire.**

|  | **Mental wellbeing (SWEMWBS)** | | | |
| --- | --- | --- | --- | --- |
|  | Multiple imputation adjusted models 1-3 (n=506) | | | Complete case analysis model 3 (n=432) |
| Variance | 17.32 (15.19-19.76) | 14.03 (12.31, 15.99) | 13.99 (12.28, 15.93) | 13.63 (11.86, 15.67) |
|  |  |  |  |  |
| Pre-transition emotional difficulties | -0.28 (-0.40, -0.17)  p<0.001 | -0.12 (-0.23, -0.00) p=0.041 | -0.12 (-0.24, -0.01) p=0.030 | -0.11 (-0.23, 0.01) p=0.062 |
| Pre-transition behavioural difficulties | -0.38 (-0.56, -0.20) p<0.001 | -0.31 (-0.47, -0.15) p<0.001 | -0.30 (-0.46, -0.13) p<0.001 | -0.29 (-0.46, -0.12) p=0.001 |
| Female | -0.33 (-1.10, 0.45) p=0.407 | -0.07 (-0.78, 0.63) p=0.840 | -0.01 (-0.72, 0.69) p=0.975 | -0.10 (-0.85, 0.65) p=0.797 |
| Family affluence scale | 0.18 (0.01, 0.35) p=0.036 | 0.18 (0.03, 0.34) p=0.018 | 0.18 (0.03, 0.34) p=0.022 | 0.19 (0.03, 0.35) p=0.021 |
| Worried secondary |  | -0.27 (-0.56, 0.02) p=0.070 | -0.28 (-0.57, 0.01) p=0.055 | -0.36 (-0.67, -0.06) p=0.020 |
| School belonging |  | 0.87 (0.43, 1.30) p<0.001 | 0.82 (0.39, 1.25) p<0.001 | 0.68 (0.20, 1.15) p=0.005 |
| Teacher cares |  | 0.78 (0.37, 1.20) p<0.001 | 0.79 (0.38, 1.20) p<0.001 | 0.93 (0.49, 1.36) p<0.001 |
| Ideas taken seriously |  | 0.88 (0.50, 1.26) p<0.001 | 0.89 (0.51, 1.27) p<0.001 | 0.90 (0.50, 1.30) p<0.001 |
| School % free school meals |  |  | -0.00 (-0.07, 0.07) p=0.989 | 0.02 (-0.06, 0.09) p=0.664 |
| Meaningfulness |  |  | 0.54 (0.05, 1.03) p=0.030 | 0.67 (0.14, 1.20) p=0.014 |

**Appendix 6: Mental wellbeing imputation models and complete case sensitivity analysis. SWEMWBS = Short Warwick Edinburgh Mental Wellbeing Scale.**

|  | **Total difficulties (SDQ)** | | |  |  |
| --- | --- | --- | --- | --- | --- |
|  | Multiple imputation adjusted models 1-3 (n=506) | | | Multiple imputation model 3 with outcome range restricted (sensitivity analysis)* | Complete case analysis model 3 (sensitivity analysis) (n=412) |
| Variance | 31.63 (27.70, 36.12) | 25.62 (22.43, 29.27) | 25.61 (22.40, 29.27) | 25.55 (22.34, 29.21) | 25.41 (21.99, 29.37) |
|  |  |  |  |  |  |
| Pre-transition emotional difficulties (M&MF) | 0.62 (0.46, 0.78) p<0.001 | 0.44 (0.28, 0.59) p<0.001 | 0.43 (0.28, 0.59) p<0.001 | 0.43 (0.27, 0.59) p<0.001 | 0.43 (0.27, 0.60) p<0.001 |
| Pre-transition behavioural difficulties (M&MF) | 0.51 (0.25, 0.78) p<0.001 | 0.39 (0.13, 0.64) p=0.003 | 0.40 (0.13, 0.66) p=0.004 | 0.40 (0.13, 0.66) p=0.004 | 0.37 (0.12, 0.61) p=0.003 |
| Female | -0.69 (-1.72, 0.35) p=0.196 | -0.86 (-1.80, 0.09) p=0.076 | -0.87 (-1.82, 0.08) p=0.072 | -0.87 (-1.82, 0.08) p=0.073 | -0.59 (-1.64, 0.45) p=0.266 |
| Family affluence scale | -0.27 (-0.50,  -0.05) p=0.018 | -0.28 (-0.48, -0.08) p=0.007 | -0.30 (-0.50, -0.09) p=0.005 | -0.30 (-0.50, -0.09) p=0.005 | -0.28 (-0.50, -0.06) p=0.013 |
| Worried secondary |  | 0.20 (-0.21, 0.60) p=0.337 | 0.21 (-0.19, 0.61) p=0.300 | 0.21 (-0.19, 0.62) p=0.293 | 0.16 (-0.27, 0.59) p=0.460 |
| School belonging |  | -0.74 (-1.32, -0.16) p=0.012 | -0.74 (-1.32, -0.15) p=0.013 | -0.74 (-1.32, -0.16) p=0.013 | -0.70 (-1.37, -0.02) p=0.043 |
| Teacher cares |  | -1.71 (-2.31, -1.12) p<0.001 | -1.71 (-2.31, -1.12) p<0.001 | -1.71 (-2.31, -1.12) p<0.001 | -1.74 (-2.36, -1.11) p<0.001 |
| Ideas taken seriously |  | -0.97 (-1.46, -0.49) p<0.001 | -0.97 (-1.45, -0.48) p<0.001 | -0.97 (-1.45, -0.48) p<0.001 | -0.92 (-1.48, -0.36) p=0.001 |
| School % free school meals |  |  | -0.05 (-0.15, 0.06) p=0.403 | -0.04 (-0.15, 0.06) p=0.406 | -0.07 (-0.17, 0.03) p=0.181 |
| Meaningfulness |  |  | -0.00 (-0.73, 0.72) p=0.993 | -0.01 (-0.73, 0.71) p=0.981 | 0.06 (-0.67, 0.79) p=0.872 |

**Appendix 7: Total difficulties imputation models and sensitivity analyses. SWEMWBS = Short Warwick Edinburgh Mental Wellbeing Scale; M&MF = Me and My Feelings Questionnaire. *Due to some imputed values being outside of the possible range (see Appendix 3), a sensitivity analysis was run with values restricted to possible range.**

|  | **Emotional difficulties (SDQ subscale)** | | |  |  |
| --- | --- | --- | --- | --- | --- |
|  | Multiple imputation adjusted models 1-3 (n=506) | | | Multiple imputation model 3 with outcome range restricted (sensitivity analysis)* | Complete case analysis model 3 (n=415) |
| Variance | 5.04 (4.42, 5.75) | 4.45 (3.90, 5.08) | 4.46 (3.91, 5.09) | 4.43 (3.88, 5.05) | 4.25 (3.71, 4.87) |
|  |  |  |  |  |  |
| Pre-transition emotional difficulties (M&MF) | 0.29 (0.23, 0.35) p<0.001 | 0.20 (0.14, 0.26) p<0.001 | 0.20 (0.14, 0.26) p<0.001 | 0.20 (0.14, 0.26) p<0.001 | 0.20 (0.14, 0.27)  p<0.001 |
| Female | 0.49 (0.06, 0.91) p=0.025 | 0.37 (-0.02, 0.77) p=0.065 | 0.35 (-0.04, 0.75) p=0.078 | 0.36 (-0.04, 0.75) p=0.078 | 0.41 (-0.00, 0.82) p=0.050 |
| FAS (secondary school) | -0.07 (-0.16, 0.02) p=0.125 | -0.06 (-0.15, 0.02) p=0.137 | -0.08 (-0.16, 0.01) p=0.079 | -0.08 (-0.16, 0.01) p=0.084 | -0.07 (-0.16, 0.02) p=0.125 |
| Worried secondary |  | 0.22 (0.01, 0.42) p=0.037 | 0.23 (0.02, 0.43) p=0.031 | 0.23 (0.03, 0.43) p=0.028 | 0.18 (0.00, 0.35) p=0.045 |
| Belong (SHRN) |  | -0.31 (-0.56, -0.06) p=0.017 | -0.31 (-0.57, -0.06) p=0.016 | -0.31 (-0.56, -0.05) p=0.018 | -0.27 (-0.54, 0.00) p=0.050 |
| Teacher care (SHRN) |  | -0.20 (-0.45, 0.06) p=0.126 | -0.20 (-0.45, 0.05) p=0.109 | -0.21 (-0.45, 0.03) p=0.080 | -0.33 (-0.58, -0.08) p=0.009 |
| My ideas (SHRN) |  | -0.47 (-0.68, -0.26) p<0.001 | -0.47 (-0.68, -0.26) p<0.001 | -0.46 (-0.67, -0.25) p<0.001 | -0.41 (-0.63, -0.18) p<0.001 |
| FSM |  |  | -0.03 (-0.07, 0.00) p=0.079 | -0.03 (-0.07, 0.00) p=0.079 | -0.04 (-0.07, -0.00) p=0.035 |
| Meaningfulness |  |  | 0.07 (-0.18, 0.32) p=0.594 | 0.06 (-0.19, 0.31) p=0.626 | 0.05 (-0.20, 0.30) p=0.695 |

**Appendix 8: Emotional difficulties imputation models and sensitivity analyses. SDQ = Strengths and Difficulties Questionnaire; M&MF = Me and My Feelings Questionnaire. *Due to some imputed values being outside of the possible range (see Appendix 3), a sensitivity analysis was run with values restricted to possible range.**

|  | **Conduct problems (SDQ subscale)** | | |  |  |
| --- | --- | --- | --- | --- | --- |
|  | Multiple imputation adjusted models 1-3 (n=506) | | | Multiple imputation model 3 with outcome range restricted (sensitivity analysis)* | Complete case analysis model 3 (n=420) |
| Variance | 2.63 (2.31, 3.00) | 2.44 (2.14, 2.77) | 2.44 (2.14, 2.78) | 2.42 (2.12, 2.75) | 2.32 (2.01, 2.68) |
|  |  |  |  |  |  |
| Pre-transition behavioural difficulties (M&MF) | 0.32 (0.25, 0.39) p<0.001 | 0.28 (0.21, 0.35) p<0.001 | 0.28 (0.21, 0.35) p<0.001 | 0.28 (0.21, 0.35) p<0.001 | 0.27 (0.21, 0.34) p<0.001 |
| Female | -0.32 (-0.62, -0.02)  p=0.039 | -0.38 (-0.67, -0.08) p=0.012 | -0.39 (-0.68, -0.09) p=0.010 | -0.38 (-0.67, -0.09) p=0.010 | -0.24 (-0.55, 0.08) p=0.137 |
| FAS (secondary school) | -0.05 (-0.12, 0.02)  p=0.159 | -0.05 (-0.11, 0.02), p=0.152 | -0.05 (-0.12, 0.02), p=0.133 | -0.05 (-0.11, 0.02), p=0.142 | -0.04 (-0.10, 0.03) p=0.284 |
| Worried secondary |  | 0.03 (-0.07, 0.14) p=0.529 | 0.04 (-0.07, 0.15) p=0.499 | 0.04 (-0.07, 0.15) p=0.480 | 0.03 (-0.09, 0.15) p=0.585 |
| Belong (SHRN) |  | -0.12 (-0.32, 0.08) p=0.232 | -0.12 (-0.32, 0.09) p=0.254 | -0.12 (-0.31, 0.08) p=0.252 | -0.07 (-0.27, 0.14) p=0.520 |
| Teacher care (SHRN) |  | -0.41 (-0.62, -0.21) p<0.001 | -0.42 (-0.62, -0.21) p<0.001 | -0.42 (-0.61, -0.22) p<0.001 | -0.48 (-0.66, -0.29) p<0.001 |
| My ideas (SHRN) |  | -0.06 (-0.22, 0.09) p=0.425 | -0.07 (-0.23, 0.10) p=0.424 | -0.06 (-0.22, 0.10) p=0.440 | -0.08 (-0.25, 0.09) p=0.338 |
| FSM |  |  | -0.01 (-0.04, 0.02) p=0.620 | -0.01 (-0.04, 0.02) p=0.642 | -0.01 (-0.04, 0.02) p=0.495 |
| Meaningfulness |  |  | -0.05 (-0.29, 0.18) p=0.655 | -0.06 (-0.29, 0.17) p=0.627 | 0.00 (-0.23, 0.23) p=0.982 |

**Appendix 9: Conduct problems imputation models and sensitivity analyses. SDQ = Strengths and Difficulties Questionnaire; M&MF = Me and My Feelings Questionnaire. *Due to some imputed values being outside of the possible range (see Appendix 3), a sensitivity analysis was run with values restricted to possible range.**

|  | Intraclass correlations (ICCs) | 95% confidence interval |
| --- | --- | --- |
| SWEMWBS | 0.059 | -0.005, 0.123 |
| SDQ total difficulties | 0.054 | -0.014, 0.123 |
| SDQ emotional difficulties | 0.024 | -0.029, 0.078 |
| SDQ conduct problems | 0.067 | -0.004, 0.139 |

**Appendix 10: Intraclass correlations for each secondary school outcome variable**
